# The EDKB: an established knowledge base for endocrine disrupting chemicals

**DOI:** 10.1186/1471-2105-11-S6-S5

**Published:** 2010-10-07

**Authors:** Don Ding, Lei Xu, Hong Fang, Huixiao Hong, Roger Perkins, Steve Harris, Edward D Bearden, Leming Shi, Weida Tong

**Affiliations:** 1 Division of Bioinformatics, Z-Tech Corporation, an ICF International Company at NCTR/FDA, USA; 2 National Center for Toxicological Research, Food and Drug Administration, Jefferson, AR 72079, USA

## Abstract

**Background:**

Endocrine disruptors (EDs) and their broad range of potential adverse effects in humans and other animals have been a concern for nearly two decades. Many putative EDs are widely used in commercial products regulated by the Food and Drug Administration (FDA) such as food packaging materials, ingredients of cosmetics, medical and dental devices, and drugs. The Endocrine Disruptor Knowledge Base (EDKB) project was initiated in the mid 1990’s by the FDA as a resource for the study of EDs. The EDKB database, a component of the project, contains data across multiple assay types for chemicals across a broad structural diversity. This paper demonstrates the utility of EDKB database, an integral part of the EDKB project, for understanding and prioritizing EDs for testing.

**Results:**

The EDKB database currently contains 3,257 records of over 1,800 EDs from different assays including estrogen receptor binding, androgen receptor binding, uterotropic activity, cell proliferation, and reporter gene assays. Information for each compound such as chemical structure, assay type, potency, etc. is organized to enable efficient searching. A user-friendly interface provides rapid navigation, Boolean searches on EDs, and both spreadsheet and graphical displays for viewing results. The search engine implemented in the EDKB database enables searching by one or more of the following fields: chemical structure (including exact search and similarity search), name, molecular formula, CAS registration number, experiment source, molecular weight, etc. The data can be cross-linked to other publicly available and related databases including TOXNET, Cactus, ChemIDplus, ChemACX, Chem Finder, and NCI DTP.

**Conclusion:**

The EDKB database enables scientists and regulatory reviewers to quickly access ED data from multiple assays for specific or similar compounds. The data have been used to categorize chemicals according to potential risks for endocrine activity, thus providing a basis for prioritizing chemicals for more definitive but expensive testing. The EDKB database is publicly available and can be found online at http://edkb.fda.gov/webstart/edkb/index.html.

**Disclaimer:***The views presented in this article do not necessarily reflect those of the US Food and Drug Administration.*

## Background

Evidence that certain man-made chemicals have the ability to disrupt the endocrine systems of vertebrates by mimicking endogenous hormones has sparked intense international scientific discussion and debate [1].  The growing national concern resulted in legislation, including the amendments of the Safe Drinking Water Act and the Federal Food, Drug and Cosmetic Act [2] and passage of the 1996 Food Quality Protection Act mandating that the Environmental Protection Agency (EPA) develop a screening program for endocrine disruptors (EDs) [3]. Under this requirement, at least 58,000 existing chemicals would be experimentally evaluated for their potential to disrupt activities in the estrogen, androgen, and thyroid hormone systems [4]. Some of the chemicals were associated with products regulated by the FDA, including plastics used in food packaging, phytoestrogens, food additives, pharmaceuticals, cosmetics, etc [5]. A battery of *in vitro* and short-term *in vivo* screening assays would be used to provide guidance for subsequent longer term, more definitive *in vivo* tests for toxicity [3]. 

Endocrine disruption is associated with interference caused by exogenous chemicals of the normal production, release, transport, metabolism, binding, action, or elimination of natural hormones in the body responsible for the maintenance of homeostasis and regulation of developmental processes [6,7]. Effects of EDs are known to occur in multiple endocrine axes such as estrogen, androgen, thyroid hormone, prolactic, and insulin systems. The putative adverse effects of EDs are wide ranging and the mechanisms of action are concomitantly diverse; many assay protocols have been used to measure their effects [8-10]. A vast body of literature has accumulated to demonstrate that suspected and known EDs are structurally diverse with many acting via binding to hormone protein receptors [11,12]. The multidimensional aspects of the science of EDs amplify the importance of a corresponding knowledge base such as the one discussed in this manuscript aggregating existing knowledge for the research and regulatory communities.

In the fall of 1996, a National Science and Technology Council [13] report on EDs identified a need for new databases and information systems. The report called for “a compilation of the results of chemicals in various short-term screening tests and *in vivo* assays to assist in the evaluation of their sensitivity, specificity and general predictiveness.”  Although these assays and tests have been performed many times by different procedures in many labs, the experimental results were scattered throughout the literature, making it difficult for researchers to find, compare, and evaluate relevant data and the assay protocols that generated the data. The Endocrine Disruptor Knowledge Base (EDKB) project, developed by the FDA’s National Center for Toxicological Research (NCTR), arose from a necessity for new information systems focused on aggregating knowledge of EDs with experimental results relevant to estrogenic, androgenic, and other ED data in one accessible location.  This collection of experimental results from diverse assays enables comparative analysis for a wide variety of chemicals and serves a basis for developing *in silico* predictive models for prioritizing potential EDs for further study. 

Online chemical toxicity databases with the capabilities of searching both chemical structure and biological activities are urgently needed for the regulatory and research community [14-16]. Two large efforts, TOXNET (TOXicology Data NETwork) and Tox21 [17-21], have been developed by government agencies focused on public database and data access. TOXNET provides free access and easy searching in a cluster of databases covering toxicology, hazardous chemicals, environmental health, and toxic releases [22]. The ChemIDplus database in TOXNET offers structural search capabilities. Tox21 is expected to deliver biological activity profiles that might enable predictive assays of *in vivo* toxicities for the thousands of poorly studied substances of concern to regulatory authorities in the United States and other countries [23]. While these two large programs will provide rich information for chemical toxicity, they do not provide domain specific knowledge for EDs. 

The EDKB project was initiated as a research asset to help address regulatory concerns on EDs. The online database provides contains chemicals spanning a wide range of FDA-regulated products including drugs, food, and cosmetics as well as EPA-regulated products such as pesticides, chemical waste, and toxic metals. The EDKB database has been used extensively for over a decade to help identify EDs, develop predictive toxicology models, and prioritize chemicals for laborious, expensive testing [4,5,12,24-26].

## Construction and content

**Figure 1:**
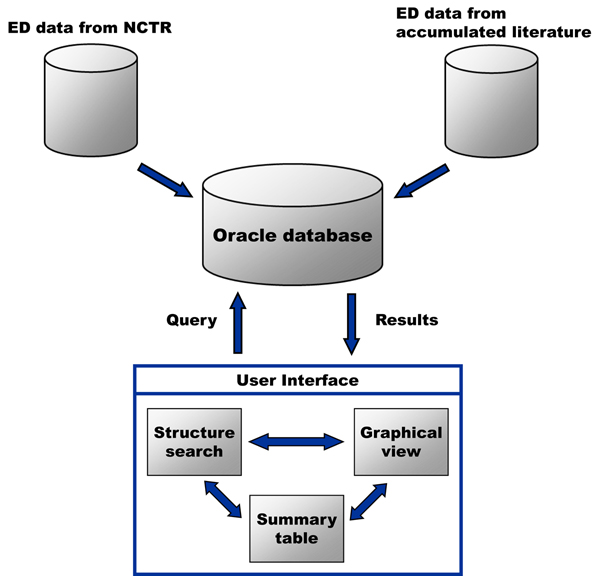
**Data flowchart for the EDKB database** In house data and literature results are stored in an ORACLE database, which can be communicated with using the interface. The user interface can link chemical knowledge in any one of its components to the other two.

The EDKB database is a client-server application consisting of a Java front-end and an ORACLE database serving as the data repository. The client application runs on the user’s workstation and allows researchers to conduct Boolean queries of the relational database and view the results. The database contains 3,257 records for over 1800 chemical compounds and will be expanded in the future. Many chemicals have data from several different assays, including data from in-house competitive binding assays (e.g., NCTR generated binding assay data for both estrogen and androgen receptors) [27-30]. The curated data are hyperlinked to the corresponding literature source in query results. Figure 1 displays a data flow model of the EDKB database.

The distribution of the data among different assay types is shown in Table 1. Endpoints were often measured as a relative activity to a reference chemical. For example, the reference chemical for estrogenic activity, 17β-Estradiol, is defined to have an activity value of 2 (log_10_100=2), while R1881 is a reference chemical with the defined activity value of 2 (log_10_100=2) for androgen receptor binding. Consequently, estrogen activity values for the EDKB chemicals range from 2.94 (strongest) to -4.5 (weakest) while androgen activity values range from 3.18 to -3.56; each covers a range of 7 orders of magnitude. Note that in the EDKB database, the activity value -10000 is assigned to inactive chemicals; additionally, chemicals that have very weak binding may be assigned placeholder values from -5 to -10000 [27,28]. 

**Table 1:** Summary of the data contained in the EDKB database

Assay type	Number of records	Standard chemical to be compared	Endpoint	Log (Activity) Range
Estrogen Receptor Binding	616	Estradiol (2.0)	*RBA	From 2.94 to -4.5
Androgen Receptor Binding	230	R1881 (2.0)	RBA	From 3.18 to -3.56
Uterotropic	1707	Estradiol (2.0)	**RP	From 3.93 to -3.44
Cell proliferation	160	Estradiol (2.0)	***RPP	From 3.0 to -4.22
Reporter gene	544	Estradiol (2.0)	RP	From 2.18 to -5.38

A summary of the distribution of data, standard chemicals to be compared, endpoints, and activity ranges.

The values in parentheses are log activity for a standard chemical

* RBA: Relative Binding Affinity

** RP: Relative Potency

*** RPP: Relative Proliferation Potency

The EDKB database has been populated with assay data from rat, mouse, and human and contains a broad chemical structure diversity. Table 2 classifies the data based on chemical structure category. Categories that contain more active records than inactive records are bolded, such as phytoestrogens, diethylstilbestrol (DES)-like chemicals, steroidal chemicals, etc. 

**Table 2:** Structure categories in the EDKB database

Structure categories	Number of records	Active /in active	Number of chemicals
Benzene	12	0/12	7
DDT	164	**101/63**	33
DES	109	**95/14**	22
Flutamide	8	**5/3**	5
NoRing	11	1/10	8
PAH	6	0/6	3
PCB	45	**37/8**	12
Pesticide	92	29/63	20
Phenol	110	**80/30**	35
Phthalate	40	14/26	8
Phytoestrogen	147	**110/37**	46
Siloxane	6	**3/3**	3
Steroid	282	**194/88**	58
Other	2225	358/1867	1610

A display of the number of records, chemicals, and active/inactive spread for each structure category.

## Utility

**Figure 2:**
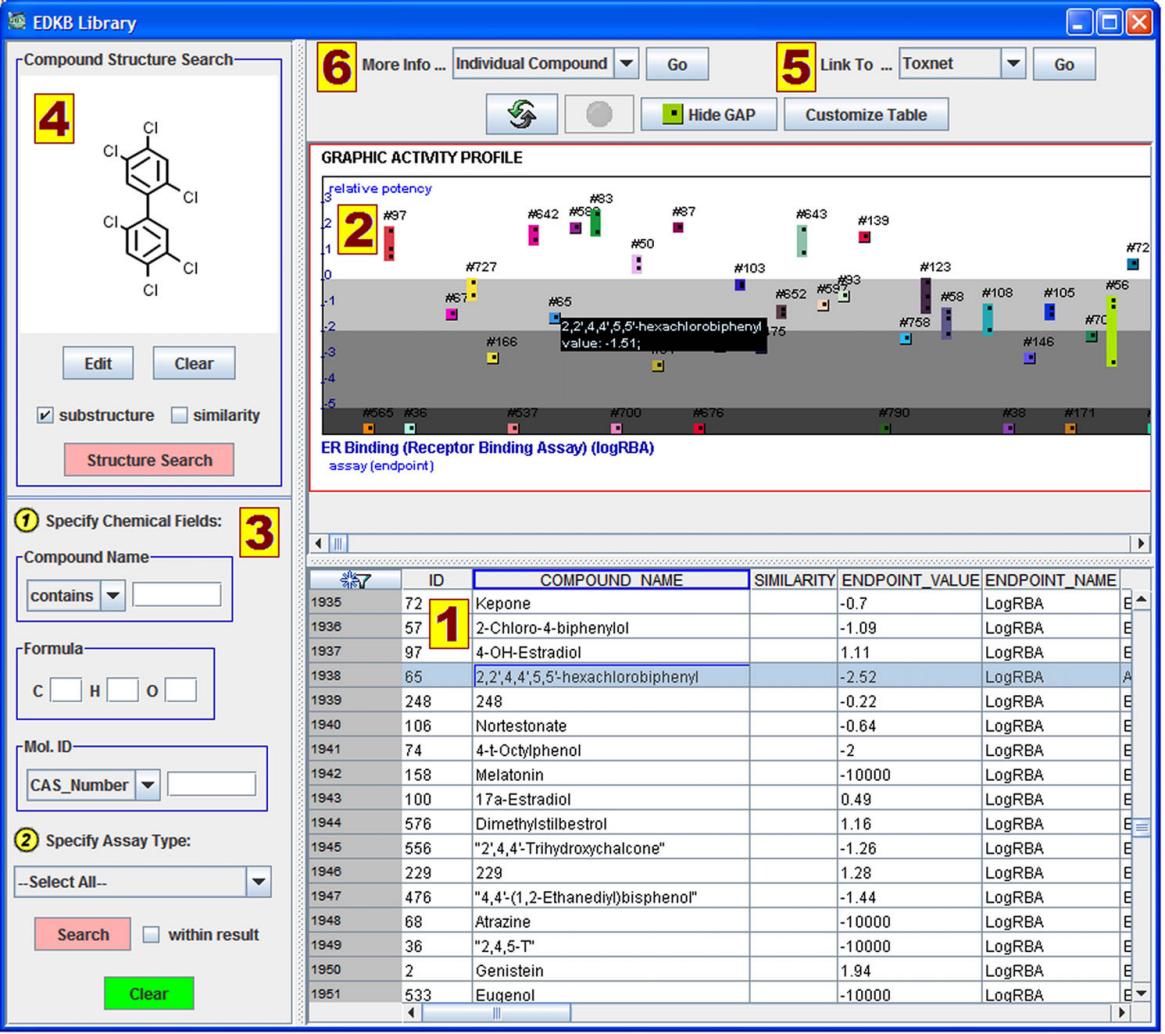
**User interface for the EDKB database** Six key components of the user interface are numbered and described in the manuscript.

The EDKB database has been online since 1997 and is still actively used by government, academic, and private sectors. It is free to use and publicly available on the internet at http://edkb.fda.gov/webstart/edkb/index.html. Six main components of the interface are labeled in Figure 2 and described below.

1. The primary component of the EDKB database is the table listing the chemical compound data. The spreadsheet format allows easy browsing of the entire database and supports column-specific sorting, searching, and filtering options. Each record contains a variety of information including name, assay type, CAS number, chemical formula, experiment source, molecular weight, etc. 

2. The Graphic Activity Profile (GAP) shows the relative potency of compounds on a log base 10 scale. Compounds observed in multiple experiments may exhibit a range rather than a single point.  The GAP table plots all data entries that are currently visible in the spreadsheet view (i.e., not hidden by filters).

3. The search panel provides a simple way to find desired chemical compounds in the EDKB database. The chemical structure can be used to locate compounds that are similar to or are substructures of the selected compound. The database can also be searched by compound name, chemical formula, various molecular IDs, and assay type. Searching within previous results is supported as well. 

4. The interface includes a graphical display of the chemical structure of any compound individually selected in the table. The Edit button opens the Molecule Sketcher, which can be used to manually edit the chemical structure or to change the notation (e.g., making H atoms explicit). After editing or creating a chemical structure, a substructure or similarity search can be performed. 

5. Compounds in the EDKB database can be directly linked to public online databases including TOXNET, Cactus, NCI DTP, etc. Using the “Link To” feature will open the user’s web browser and automatically search the selected website based on the appropriate identifiers, which can save significant amounts of time. 

6. A detailed summary of any individual compound can be opened in a new window by using the “More Info” button. This functionality is useful to summarize all the available information for this chemical, such as synonyms, relevant experiment details, and references. Additionally, each experiment involving the compound has a summary page that can be accessed from here. 

## Results and discussion

**Figure 3:**
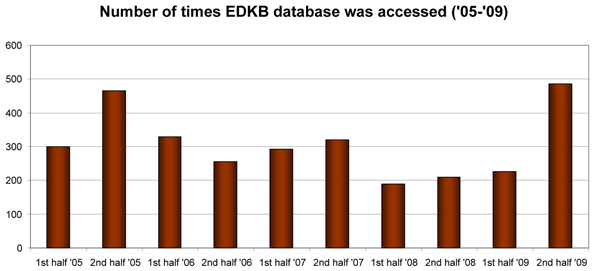
**User statistics of the EDKB database** Bar graph displaying the number of times the EDKB database was accessed per half year between 2005 and 2009.

The EDKB database has users from government, academia, and private sectors throughout the world. Recent user statistics, shown in Figure 3, indicate that the database has been steadily accessed by a significant number of users over the past five years. We will show three use cases for the EDKB database to assess the estrogenic activity potential of three interesting chemicals from among 58,000 compounds that the EPA chose for screening for ED activity [4]: genistein, L-ascorbic acid, and 4,4’,4”-ethylidynetrisphenol. See additional file 1 for the data used to perform the analysis in this section.

Genistein, also known as 5,7,4'-trihydroxyisoflavone, is a phytochemical that can be found in soybean-derived food products. Searching for genistein by compound name returned 14 records in the EDKB database, all of which showed estrogenic activity as compared to the standard endogenous sex hormone 17β-estradial. The EDKB database shows that genistein has a relatively high binding affinity for the estrogen receptor (ER) nuclear protein. However, genistein results have considerably lower endpoint values relative to 17β-estradial in reporter gene assays measuring ER transcription factor activity, and lower still relative values in *in vitro* assays of cancer cell proliferation. In uterotrophic assays measuring uterine weight gain, genistein is some 100,000 fold less potent than 17β-estradial.  Based on this data alone, genistein could be a potent ED that competitively binds ER in a similar manner to 17β-estradial. It is possible that genistein mimics the sex hormone sufficiently to cause down regulation of ER, resulting in suppression of ER regulated mRNA. Thus, genistein is likely an ED and substantial further testing is warranted.

L-ascorbic acid, also known as Vitamin C, is an essential nutrient for humans and certain other animal species. The ED data for this chemical is not available in the EDKB. Thus, we conducted the structure similarity search by comparing its chemical structure with the compounds in the EDKB. We found that the 10 chemicals (occurring in 14 records) with the most similar structures (40 to 50%  similarity) have all been measured as inactive in estrogenicity assays. Accordingly, L-ascorbic acid could be assigned a low priority for further testing as a potential endocrine disrupting chemical.

The chemical 4,4’,4”-ethylidynetrisphenol is used as a cross linking or branching agent in various polymer applications, such as use in polycarbonates, epoxies, adhesives, coatings, and antioxidants [31].  While no name matches were found for this chemical in the EDKB, the same structure search strategy mentioned above was applied, returning four compounds with a similarity rating of 100% as well as several others with very high similarity ratings. Among the top ten most similar compounds, a majority of the 45 recorded instances show estrogenic activity. These results indicate that 4,4’,4”-ethylidynetrisphenol is a potential ED and could be considered for further testing.

These use cases illustrate that once the database is established, queries enable knowledge-based conclusions that can lead to research hypotheses and questions to be posed for regulatory decision-making. 

## Conclusion

In an age of information technology, it is crucial to have a database containing specific toxicology data and structure search capabilities. The EDKB database fulfills this role and is valuable in extending predictive systems to real-world regulatory implementations. It is freely available on the web and assists researchers in accessing and interpreting ED data.

## List of abbreviations used

ED(s): Endocrine Disruptor(s); EDKB: Endocrine Disruptor Knowledge Base; EPA: Environmental Protection Agency; ER: Estrogen Receptor; FDA: Food and Drug Administration; GAP: Graphic Activity Profile; NCTR: National Center for Toxicological Research

## Authors’ contributions

DD created the first draft of the manuscript. LX performed data analysis. HF and HH coordinated data analysis and manuscript writing. LX, HF, HH, and RP helped significantly to draft the manuscript. SH performed the software and database programming. LS helped the development of the EDKB database. WT helped coordinate the project and finalized the manuscript. EDB coordinated EDKB project components aimed at integration with the FDA Janus data warehouse.  All authors have read and approved the final manuscript.

## Competing interests

The authors declare that they have no competing interests.

## Supplementary Material

Additional file 1 The first table in the file gives an overview of the 3 chemicals: genistein, L-ascorbic acid, and 4,4’,4”-ethylidynetrisphenol. The second displays the results when the EDKB database was searched by compound name for genistein. The third and forth display results using the compound structure similarity for L-ascorbic acid and 4,4’,4”-ethylidynetrisphenol, respectively. Click here for file
