# Supplementary material for: The EDKB: an established knowledge base for endocrine disrupting chemicals
Source: BMC Bioinformatics. 2010 Oct 7;11(Suppl 6):S5. doi: 10.1186/1471-2105-11-S6-S5 (PMC3026379; doi:10.1186/1471-2105-11-S6-S5)
Supplement: Additional file 1 — The first table in the file gives an overview of the 3 chemicals: genistein, L-ascorbic acid, and 4,4’,4”-ethylidynetrisphenol. The second displays the results when the EDKB database was searched by compound name for genistein. The third and forth display results using the compound structure similarity for L-ascorbic acid and 4,4’,4”-ethylidynetrisphenol, respectively. [file 1471-2105-11-S6-S5-S1.PDF]

EDKB database results for 3 specific chemicals

Three chemicals from EPA’s dataset with 58,000 compounds

Structure	Formula	Name	Contained in EDKB	Active/inactive
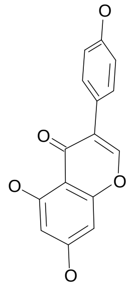	$C_{15}H_{10}O_5$	Genistein	Yes	14/0
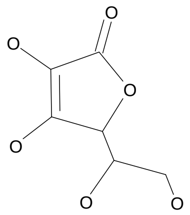	$C_6H_8O_6$	L-ascorbic acid	No	0/14 (by similarity search)
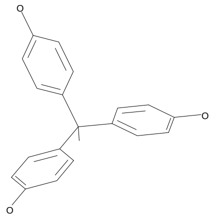	$C_{20}H_{18}O_3$	4,4’,4”-ethylidynetrisphenol	No	40/5 (by similarity search)

The 14 activity results found in the EDKB database for genistein

Chemical	Endpoint value	Endpoint name	Assay name
Genistein	-1.85	LogRPP	Escreen (Cell Proliferation Assay)
Genistein	-0.52	LogRP	ER Gene (Reporter Gene Assay)
Genistein	-1.31	LogRP	ER Gene (Reporter Gene Assay)
Genistein	-1.85	LogRPP	Escreen (Cell Proliferation Assay)
Genistein	-2.44	LogRBA	AR Binding (Receptor Binding Assay)
Genistein	0.7	LogRBA	ER Binding (Receptor Binding Assay)
Genistein	0.6	LogRBA	ER Binding (Receptor Binding Assay)
Genistein	1.94	LogRBA	ER Binding (Receptor Binding Assay)
Genistein	-0.17	LogRBA	ER Binding (Receptor Binding Assay)
Genistein	1.56	LogRBA	ER Binding (Receptor Binding Assay)
Genistein	-0.36	LogRBA	ER Binding (Receptor Binding Assay)
Genistein	-2.7	LogRP	Uterotrophic Assay
Genistein	-2.96	LogRP	Uterotrophic Assay
Genistein	-2.4	LogRP	Uterotrophic Assay

The top 10 similar chemicals found in the EDKB database for L-ascorbic acid

Query	Top 10 Similar Chemicals		
Structure	Similarity	Structure	Active/inactive
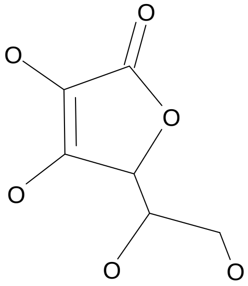	0.49	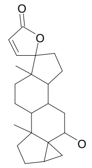	0/2
	0.47	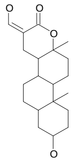	0/1
	0.47	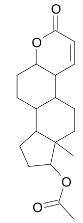	0/1
	0.47	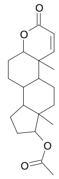	0/2
	0.43	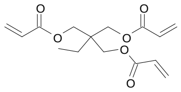	0/1
	0.43	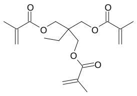	0/1
	0.42	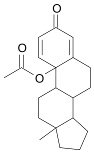	0/2
	0.42	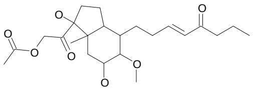	0/1
	0.41	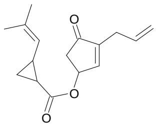	0/1
	0.41	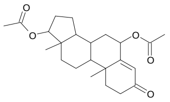	0/2
Total active/inactive records			0/14

The top 10 similar chemicals found in the EDKB database for 4,4’,4”-ethylidynetrisphenol

Query	Top 10 Similar Chemicals		
Structure	Similarity	Structure	Active/inactive
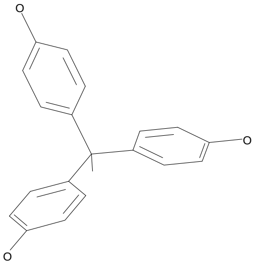	1	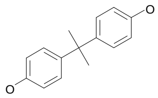	14/1
	1	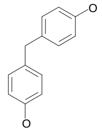	4/0
	1	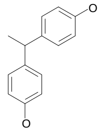	2/0
	1	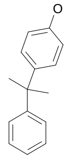	2/0
	0.9	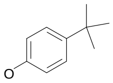	6/2
	0.9	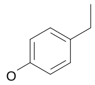	2/2
	0.88	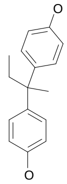	5/0
	0.88	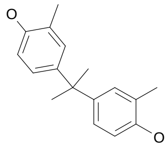	3/0
	0.88	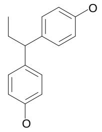	2/0
	0.88	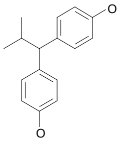	0/1
Total active/inactive records			40/5
